# Magnetic resonance‐based biomarkers in nonalcoholic fatty liver disease and nonalcoholic steatohepatitis

**DOI:** 10.1002/edm2.134

**Published:** 2020-04-20

**Authors:** Cyrielle Caussy, Lars Johansson

**Affiliations:** ^1^ Univ Lyon CarMen Laboratory INSERM INRA INSA Lyon Université Claude Bernard Lyon 1 Pierre‐Bénite France; ^2^ Hospices Civils de Lyon Département Endocrinologie Diabète et Nutrition Hôpital Lyon Sud Pierre‐Bénite France; ^3^ Antaros Medical Bioventure Hub Mölndal Sweden

**Keywords:** elastography, hepatic steatosis, magnetic resonance imaging, NAFLD, NASH

## Abstract

Nonalcoholic fatty liver disease is a growing epidemic affecting 30% of the adult population in the Western world. Its progressive form, nonalcoholic steatohepatitis (NASH), is associated with an increased risk of advanced fibrosis, cirrhosis and liver‐related mortality. Therefore, the detection of NAFLD and risk stratification according to the severity of the disease is crucial for the management of patients with NAFLD. Liver biopsy for such risk stratification strategies is limited by its cost and risks; therefore, noninvasive alternatives have been developed. Among noninvasive biomarkers developed in NAFLD, magnetic resonance (MR)‐based biomarkers have emerged as key noninvasive biomarkers in NAFLD with the ability to accurately detect hepatic steatosis and liver fibrosis. The potential utility of MRI for the detection of NASH and functional liver assessment has also recently emerged. In the current review, we will discuss the data supporting the utility of MR‐based biomarker for the detection of features of NAFLD and its potential use in clinical practice and clinical research in NAFLD.

## Graphical abstract

This paper reviews the currently available MRI‐based techniques for assessing NAFLD and NASH.

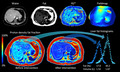

## 1 INTRODUCTION

Nonalcoholic fatty liver disease (NAFLD) is now considered as one of the most prevalent aetiologies of chronic liver disease worldwide affecting approximately 30% of the adult population.^1^, ^2^ NAFLD encompasses a spectrum of severity from a simple accumulation of fat in the liver to nonalcoholic steatohepatitis (NASH) considered as the progressive form with higher risk to progress to advanced fibrosis or cirrhosis^3^ which is associated with a higher risk of liver‐related mortality.^4^ In addition, NASH‐related cirrhosis is currently the second leading indication for liver transplants in the United States.^5^, ^6^, ^7^ Therefore, the detection of NAFLD and risk stratification according to the severity of the disease is crucial for the management of patients with NAFLD and pharmacological therapy for NASH has become an intensive field of research with promising new therapies under development.^8^, ^9^

As NASH is a disease defined by biopsy, any noninvasive imaging biomarker will per definition be compared to the biopsy‐defined characteristics of NASH, that is steatosis, inflammation and ballooning but also stage of fibrosis.^10^ However, this expensive and invasive procedure is not applicable for the screening of NASH and liver fibrosis at the level of high‐risk population or to assess longitudinal change in NASH or liver fibrosis. In addition, liver biopsy is limited by sampling error and significant inter‐ and intra‐observer variability.^11^, ^12^, ^13^, ^14^, ^15^ Therefore, noninvasive, precise, reproducible, accurate surrogates are needed for the detection of the different stage of NAFLD including NAFLD, NASH or liver fibrosis and to monitor changes in stage of the disease over time.

Several noninvasive biomarkers of NAFLD assessment including serum biomarkers, clinical predictor rules or imaging‐based measurements have been developed.^16^, ^17^ Magnetic resonance (MR)‐based biomarkers have emerged as key noninvasive biomarkers in NAFLD encompassing several modalities that enables accurate assessment for hepatic steatosis quantification or liver fibrosis assessment. Since NASH is defined by biopsies this limits the clinical utility of functional liver tests since they will per definition not compare directly with the static biopsy derived information. However, emerging data suggest that functional liver imaging may also play a role, especially in the development of new treatments for NASH, especially when the mode of action of these drugs can better be reflected by change in liver function. In the current review, we will discuss the data supporting the utility of MR‐based biomarker for the detection of features of NAFLD and its potential use in clinic or clinical research in NAFLD. The methods are summarized in Table 1.

**TABLE 1:** Magnetic resonance‐based modalities available for the assessment of NAFLD

	MRS	MRI‐PDFF	MRE	T1 (cT1)	Gadoxetate
Proposed measure of	Fat/water ratio	Fat/water ratio	Stiffness	Extracellular water	Hepatocyte function
Characteristics of NAFLD assessed	Hepatic steatosis	Hepatic steatosis	Fibrosis	Fibrosis, inflammation	N/A
Validated versus histology in humans	Yes	Yes	Yes	Yes	N/A
Proven detection of longitudinal changes in humans	Yes	Yes	Yes	Yes	No
Proven that change in end‐point reflect change in biopsies	Yes	Yes	Preliminary data that need validation	No	N/A
Functional readout	No	No	No	No	Yes

Abbreviations: MRE, magnetic resonance elastography; MRI, magnetic resonance imaging; MRS, magnetic resonance spectroscopy; N/A, not available; NAFLD, nonalcoholic fatty liver disease; PDFF, proton density fat fraction.

## 2 MR‐BASED BIOMARKER FOR THE DETECTION OF HEPATIC STEATOSIS

### 2.1 Magnetic resonance spectroscopy

The presence of NAFLD is defined by the presence of hepatic steatosis ≥5% either by imaging or histology.^18^ MRS noninvasively measures proton signals as a function of their resonance frequency. The signal intensity at frequencies corresponding to water or fat can be quantified, and the fat‐signal fraction can be calculated. MRS is highly sensitive for the detection and quantification of even small amounts of liver fat, and MRS is considered as the most accurate noninvasive method to quantify liver fat.^19^, ^20^, ^21^ However, the use of MRS in clinical practice is limited as it is not available on all clinical scanners and requires dedicated spectroscopic sequences and time‐consuming postprocessing analysis. In addition, MRS is restricted in spatial coverage owing to the volume selection required limiting measurements to a small portion of the liver. All these limitations impede the use of MRS for longitudinal monitoring.

### 2.2 Magnetic resonance imaging

As MRS, magnetic resonance imaging exploits the difference of the resonance frequencies between water and fat proton signals. MRI‐proton density fat fraction (PDFF) take into account several confounding factors that may affect the MRI estimation of tissue fat concentration for an accurate quantification of hepatic steatosis.^22^ PDFF is defined as the ratio of the density of mobile protons from triglycerides and the total density of protons from mobile triglycerides and mobile water. MRI‐PDFF is a quantitative imaging biomarker that enables accurate, repeatable and reproducible quantitative assessment of liver fat over the entire liver.^23^, ^24^, ^25^, ^26^, ^27^ Fundamental difference between histologic and MRI‐PDFF assessment of hepatic steatosis relies on the feature measured by each modality. Histological assessment estimates the number of steatotic cells in the liver, while MRI‐PDFF estimates the overall percentage of MRI‐visible protons on fat molecules in the liver.^28^ Therefore, as the fat content of a cell does not generally exceed 50%, MRI‐PDFF percentages are almost always less than half the value derived by histology.

MRI‐PDFF is well validated using magnetic resonance spectroscopy (MRS) as reference^27^, ^29^, ^30^, ^31^, ^32^ and against histology‐proven steatosis grade.^21^, ^26^, ^33^, ^34^ In a recent meta‐analysis of 23 studies with 1,679 participants, MRI‐PDFF was shown to have excellent linearity, bias and precision across different reconstruction methods, and MR scanners of different field strength and manufacturer.^35^

Advantages of MRI‐PDFF are to rapidly assess PDFF over the entire liver in a short breath hold (~20 seconds). PDFF maps are automatically reconstructed without user input or postprocessing. In addition, MRI‐PDFF methods are FDA approved and are commercially available on the three major MRI vendors, GE Healthcare, Siemens and Philips, ensuring potential widespread availability.

**FIGURE 1:**
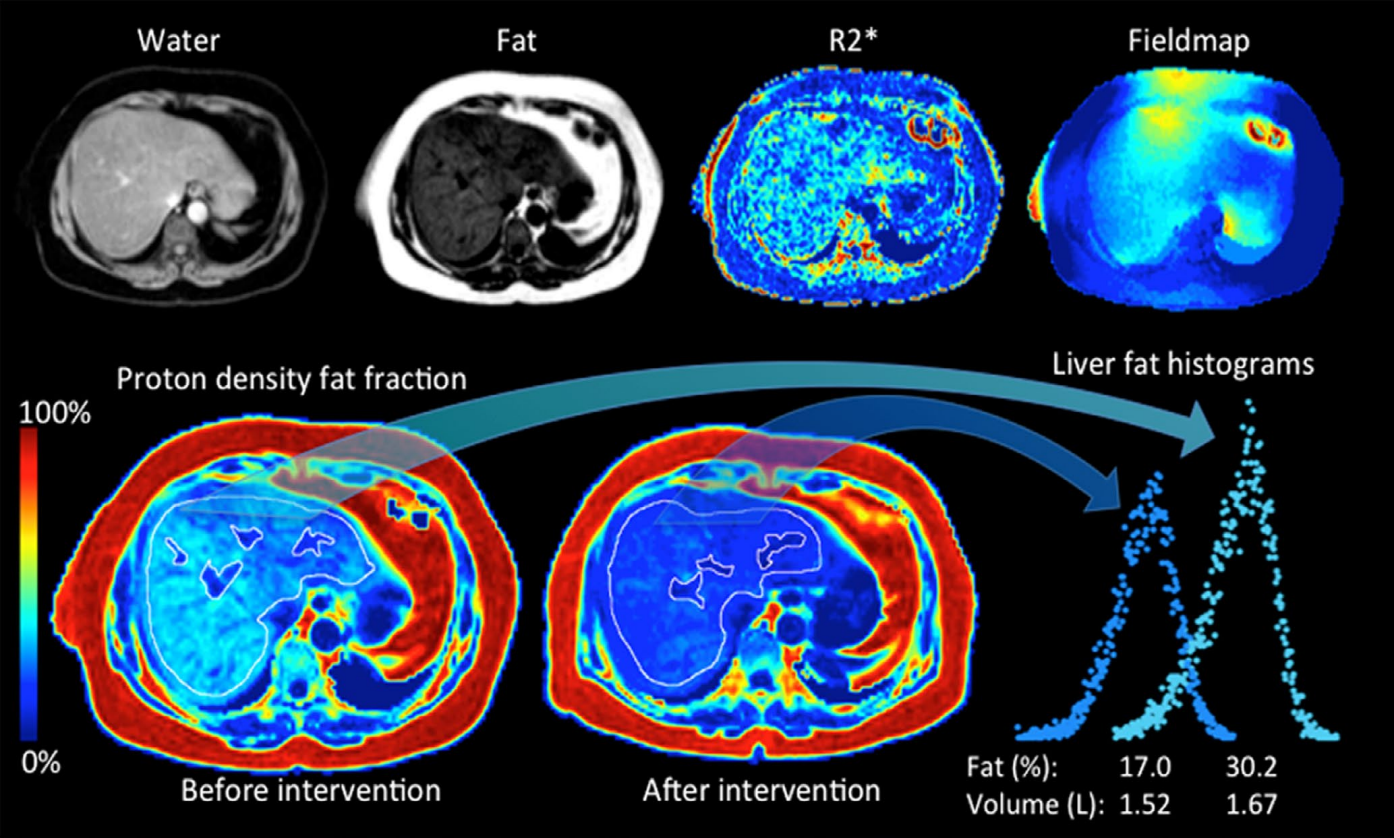
An example of a MRI‐PDFF map from the same patient before and after intervention. In this case, the entire liver is measured excluding major bile ducts and veins to improve precision. Here, also the liver volume was measured and reductions both in PDFF and liver volume are seen. The whole liver analysis also enables the histogram display and analysis of pixels throughout the liver as shown in the figure

Studies have also demonstrated the ability of MRI‐PDFF to detect longitudinal change in liver fat content.^26^, ^36^, ^37^ Indeed, two studies performed in adults and children with known or suspected NAFLD have shown that longitudinal change in MRI‐PDFF agrees closely with longitudinal change in MRS.^38^, ^39^ MRI‐PDFF has been utilized in several early phase II studies to monitor longitudinal changes in hepatic fat in patient with NAFLD^40^, ^41^ and has been implemented in over 50 intervention studies in NAFLD or NASH. An example of a PDFF map from the same patient before and after treatment is shown in Figure 1. In this case, the liver volume was also assessed since this can be of importance in understanding unexpected effects from interventional studies. An example of that is the increase in liver volumes induced by, for example, fenofibrates.^42^ MRI‐PDFF has thus emerged as noninvasive imaging biomarker suitable as an end‐point in clinical trial in NASH for internal decision‐making.^43^ For regulatory purposes in phases 2B and 3, biopsies are however still required. Finally, MRI‐PDFF has been associated with longitudinal change in histologic feature of NAFLD including NASH and liver fibrosis. Patel et al^44^ have shown that the relative reduction of liver fat quantified by MRI‐PDFF is associated with a histologic response in NASH. Finally, preliminary data have also suggested a prognostic value of MRI‐PDFF in NAFLD progression. Ajmera et al have shown that baseline MRI‐PDFF fat content is associated with longitudinal progression of fibrosis in patients with biopsy‐proven NAFLD.^45^ These preliminary data need to be validated in larger independent cohorts.

Overall, MRI‐PDFF is emerging as one of the leading noninvasive quantitative biomarkers for the quantification of hepatic steatosis in term of accuracy, precision and reproducibility. Although its cost is a limitation for a use in routine clinical practice, its utility is valuable in the context of clinical trials and may also be useful as a prognostic factor of progression of regression of NAFLD in future.

## 3 MR‐BASED BIOMARKER FOR THE DETECTION OF LIVER FIBROSIS

### 3.1 Magnetic resonance elastography

The detection and staging of liver fibrosis are important for the management of patients with NAFLD. Several studies have demonstrated that the presence of liver fibrosis is the most important predictor of mortality in NAFLD and the risk of liver‐related mortality increases exponentially with increase in fibrosis stage.^4^, ^46^, ^47^ The liver stiffness is closely correlated with stage of fibrosis and is commonly used to noninvasively quantify liver fibrosis. Several imaging‐based biomarkers have been developed including ultrasound‐based methods (reviewed in another article of this issue) and MR elastography (MRE).^48^

**FIGURE 2:**
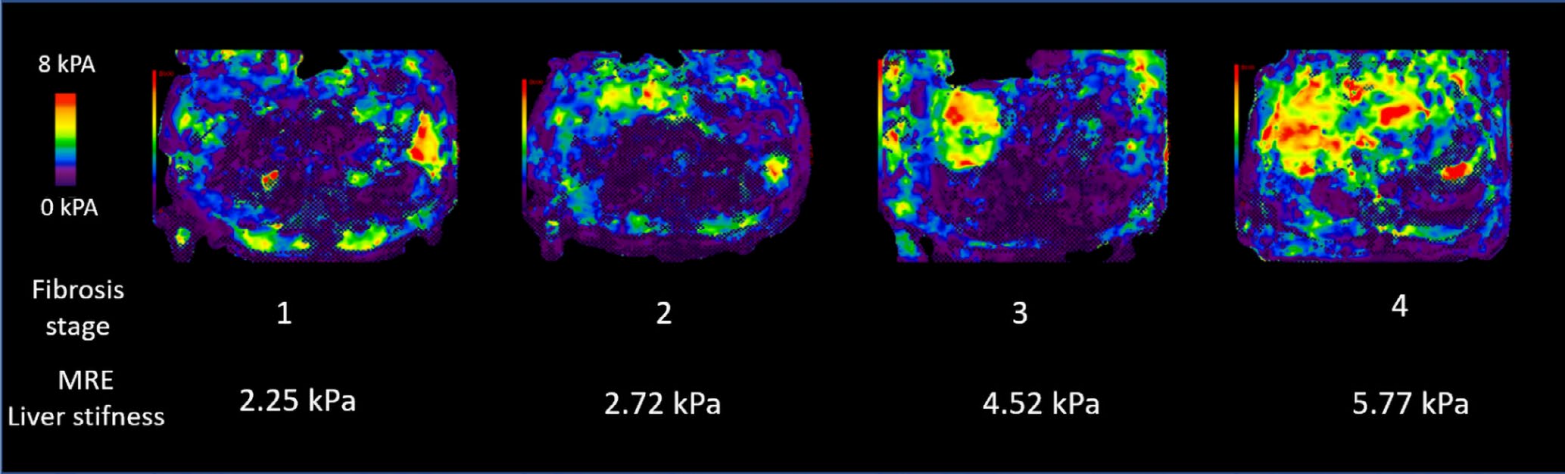
Quantitative elastograms from 4 different patients with biopsy determined fibrosis stages from 1 to 4. The figure shows a slice from each patient and MRE liver stiffness is displayed below the fibrosis stage

MRE is an MRI‐based technique that images the propagation of acoustic shear waves in the liver and applies a mathematical algorithm to compute cross‐sectional images displaying the magnitude of the complex shear modulus of liver tissue.^49^, ^50^, ^51^ MRE requires a special commercially available software and hardware (Resoundant, Inc) that can be implemented on conventional MRI scanners. During a MRI scan, a standard 60‐Hz shear wave is generated by an acoustic passive driver attached to the body wall anterior to the liver and coupled with an acoustic active driver outside the MRI room. The resulting shear waves are then visualized using a 2D gradient‐recalled‐echo pulse sequence. Noncontiguous axial slices (each roughly 10 mm thick) are acquired during 16‐second breath hold through the widest transverse section of the liver with short recovery times in between. The mean liver stiffness is a function of the average per‐pixel stiffness measurements from regions of interest in at least four axial slice locations (Figure 2).^49^, ^50^, ^51^

MRE has been reported to provide significantly higher diagnostic accuracy for the detection of advanced fibrosis in patients with NAFLD compared to clinical prediction rules including NAFLD Fibrosis Score, FIB‐4 and APRI.^52^ In addition, MRE generally outperforms all ultrasound‐based modalities and have lower risk of failure than ultrasound‐based elastography especially when BMI increases.^53^, ^54^, ^55^ The failure rate of MRE in patient with NASH is low approximately of 7.7% based upon a large cohort over 600 patients with NASH^56^ and may be due to hepatic iron overload or hepatic inflammation and chronic passive congestion.^50^ Recent head‐to‐head comparison between different imaging elastography modalities using liver biopsy as reference standard has demonstrated that MRE has higher diagnostic performance than acoustic radiation force impulse (ARFI)^54^ or vibration control transient elastography (VCTE).^49^, ^57^, ^58^ A recent meta‐analysis using individual data of 230 patients with biopsy‐proven NALFD have demonstrated that MRE has a higher diagnostic accuracy compared to VCTE for the detection of each individual stages of fibrosis.^55^ This study provides also optimal thresholds of MRE of the detection of individual stage of fibrosis that are crucial for interpretation of the results. Interestingly, the individual patient meta‐analysis confirmed the optimal threshold for the detection of advanced fibrosis (stage F3 and F4) ≥3.62 kPa with an excellent diagnostic accuracy (AUROC of 0.93) previously reported in an independent cohort by Loomba et al.^59^ The sensitivity of MRE to discriminate between lower stage of fibrosis F0 versus F1‐4 was lower, and further studies with larger multicentre cohort are needed to confirm the optimal thresholds and diagnostic performance for the detection of individual stage of fibrosis.

### 3.2 Longitudinal assessment of NAFLD using magnetic resonance elastography

Emerging data from longitudinal studies are arising and have helped to understand the potential use of MRE for longitudinal assessment of progression or regression of NAFLD. A few clinical trials in NASH have reported longitudinal changes in MRE.^60^, ^61^, ^62^, ^63^ A study by Patel et al have shown that an MRE liver stiffness reduction higher than 15% is observed in patient with BMI reduction of 5% over 24 weeks in a setting of a clinical trial.^62^ A post hoc analysis from a 24‐week phase 2 clinical trial in NASH of selonsertib is to date the only study reporting longitudinal change in MRE in patients with NASH and stage of fibrosis F2 and F3 with paired liver biopsy.^61^ This study has reported that the reduction in liver stiffness by MRE was predictive of fibrosis stage improvement with an AUROC of 0.62 (95% CI 0.46‐0.78) and predictive of NAS score improvement with an AUROC of 0.66 (95% CI 0.48‐0.83).^61^ Another post hoc analysis of the selonsertib trial has reported that a decrease in MRE ≥ 15% was associated with improvement of several markers of liver fibrosis.^64^ In line with these findings, a recent study by Ajmera et al including 102 patients with NAFLD that underwent repeated liver biopsy and paired MRE has reported that a 15% increase in liver stiffness was significantly associated with increased odds of histologic fibrosis progression.^65^ However, these preliminary data performed on a small sample size need to be confirmed and further studies are needed to determine whether a clinically meaningful change in MRE‐derived liver stiffness can predict histological treatment response or liver fibrosis progression in NASH.

Recent studies have shown that 3D MRE may be better than 2D MRE. 3D MRE is a more advanced version of the technology that can image shear‐wave fields in 3 dimensions of the entire liver rather than a smaller area of interest in the case of 2D MRE. Loomba and colleagues showed that the AUROC for diagnosing advanced fibrosis was 0.981 for 3D MRE at 40 Hz versus 0.921 for 2D MRE at 60 Hz (standard shear‐wave frequency available clinically).^66^ 3D MRE has been utilized in the setting of treatment response assessment in NASH but it requires significant expertise and is not ready for routine clinical practice.^36^

Overall, the current data available suggest that among elastography modalities, MRE is the most reliable and accurate technique for the noninvasive assessment of liver fibrosis especially in intention to diagnose compared to ultrasound‐based modalities such as ARFI and VCTE and clinical prediction rules. However, inconvenient of MRE is its cost and availability that limit its use in routine clinical practice in large population. MRE could be considered for specific cases with high BMI or when unreliable VCTE reading in second intention. Further data are needed to determine the place of each modality and to develop optimal and cost‐effective algorithm using step wise approaches for the assessment of liver fibrosis.

## 4 OTHER MR‐BASED BIOMARKER FOR THE DETECTION OF NAFLD AND NASH: T1, CORRECTED T1 AND MULTIPARAMETRIC MRI

There are currently no direct methods clinically available to assess disease activity including ballooning and inflammation in NASH. There are however several MRI methods that have been suggested to be related to disease activity and fibrosis such as magnetization transfer contrast and diffusion‐weighted imaging and T1 mapping, where T1 mapping is most widely used method today. T1 mapping of liver disease was described already in 1981^67^ and has also more recently been described for assessing liver fibrosis in cirrhotic patients.^68^ It was also proposed that T1 is a representation of extracellular fluid in the liver by Banerjee et al^69^ but that iron content of the liver will confound T1 measurements. The proposed solution was to quantify R2* (1/T2*), which is dependent on liver iron, and correct the measured T1 values by R2* to obtain a more correct assessment of T1, this is the so called corrected T1 (cT1). This study included 79 patients with various background (31 had steatohepatitis, alcoholic and nonalcoholic and 31 had viral hepatitis). Using cT1, it was shown that it was possible to discriminate between various degrees of fibrosis using Ishak fibrosis stage as reference, especially between patients without fibrosis versus those with fibrosis, and there was however no difference between patients with F1‐2 versus F3‐4. In a follow‐up study in 71 subjects with suspected NAFLD,^70^ it was also shown that cT1 could discriminate between groups with different activity scores. It should however be noted that an overlap between individual groups is present, like that of fibrosis assessment with MRE. In addition, data on the prognostic value of cT1^71^ are available but these data are originating from small sample sizes and larger studies are therefore warranted. cT1 is currently being deployed in the UK‐Biobank study,^72^ so outcome data from much larger populations can be expected in the future.

There are also some questions to be addressed using T1 and cT1 to monitor longitudinal changes in NAFLD and NASH. A potential issue is the fluctuating stores of liver glycogen, glycogen binds large amounts of water.^73^ This means that any intervention that alters liver glycogen stores also can induce changes in T1 independent of inflammation and fibrosis. Another potential issue is the use of R2* for correction of T1 since there is a strong dependency of R2* on liver fat as shown recently by Bashir et al.^74^ In fact, in this study it was shown that liver fat is the most influential covariate of hepatic R2* both at 1.5 and 3T. This means that any intervention inducing a change in liver PDFF also will induce a change in cT1 owing to the change in R2*. It can of course still be so that reductions in inflammation and fibrosis can occur and induce changes in cT1, but interventional data need to be considered in the light of changes in hepatic PDFF. In addition, the Bashir paper showed there were only very limited number of subjects that suffered from abnormally high R2* and hence very few subjects that require correction. Furthermore, the relationship between hepatic PDFF and fibrosis is not linear but rather biphasic,^57^ meaning that it is the intermediate fibrosis stages that have the highest levels of PDFF and hence are most susceptible to changes in PDFF and therefore changes in R2* inducing further complexities in interpreting data. This multiparametric liver MRI is currently developed as Liver*MultiScan* (Perspectum Diagnostics), and studies in large cohorts of patients with biopsy‐proven NAFLD are needed in order to determine the diagnostic performance for the detection of NASH and utility in longitudinal follow‐up.

Finally, combination of multifrequency 3D‐MRE including the damping ratio at 40 Hz and shear stiffness at 60 Hz combined with MRI‐PDFF had the ability to predict the presence of NASH and disease activity assessed by NAS in a cross‐sectional study design performed in obese patients undergoing bariatric surgery.^75^ Further studies are warranted to validate these results in other cohorts of patients with NASH.

## 5 ASSESSMENT OF LIVER FUNCTION BY MRI

**FIGURE 3:**
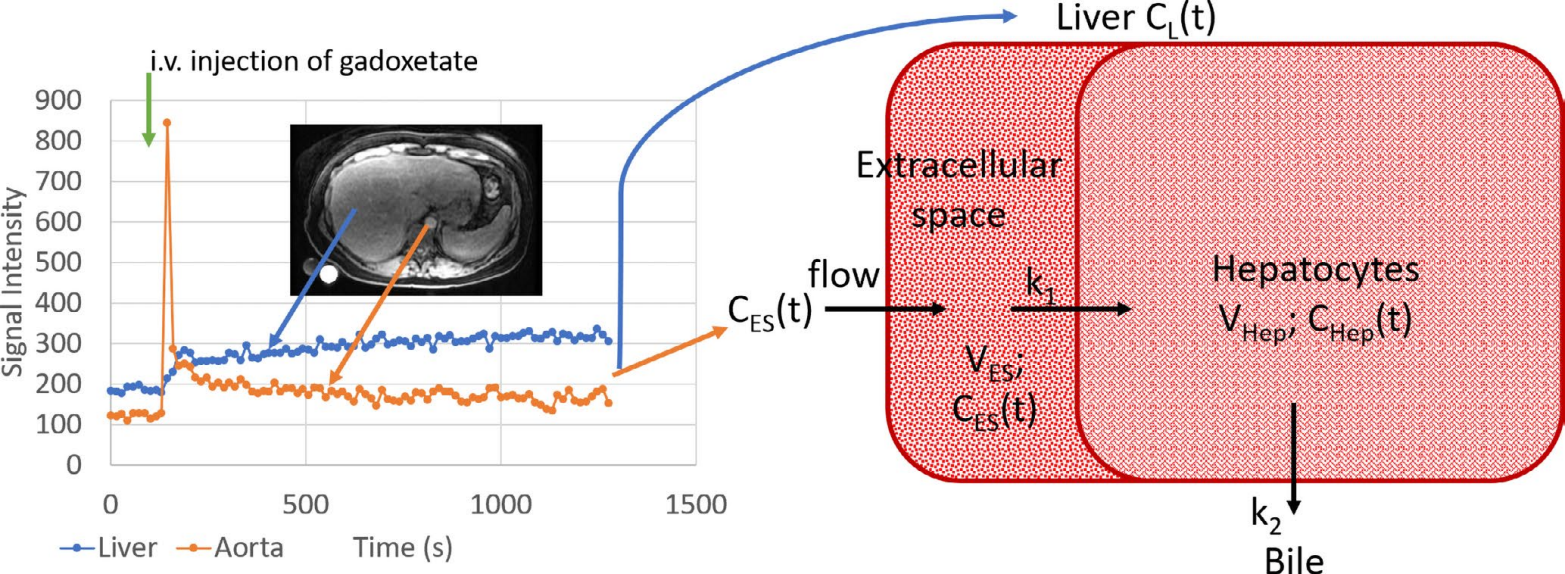
Displayed is an example of dynamic gadoxetate disodium imaging. The time‐intensity curves represent the signal in the aorta and the liver parenchyma. To the right is shown the compartmental modelling used

In most other disease areas, circulating biomarkers or imaging biomarkers that define the disease are available such as HbA1c in type 2 diabetes (T2D), creatinine‐based glomerular filtration rate (GFR) in chronic kidney disease and NT‐proBNP and ejection fraction in heart failure. There are functional tests used in combination with stressors to challenge the organ of interest for improved diagnosis and treatment monitoring, for example adenosine stress testing in coronary artery disease, glucose challenge in T2D, captopril tests in renal disease. In NAFLD and NASH, the disease is as previously mentioned defined by biopsy and hence the development of functional imaging tests has not been pushed through development to the same extent as in other disease areas and to an even lesser extent functional stress tests of the liver. There are however emerging MRI‐based techniques to study liver function. One of them is based on the use of the liver‐specific MRI contrast agent gadoxetate disodium Figure 3. Gadoxetate disodium is injected intravenously and is taken up in hepatocytes via the organic anion‐transporting polypeptide 1 (OATP1) transporter and excreted into the bile via the multidrug resistance‐associated protein 2 (MRP2) transporter. The use of gadoxetate disodium is indicated for intravenous use in T1‐weighted MRI of the liver to detect and characterize lesions in adults with known or suspected focal liver disease. Its mechanism is that normal hepatocytes take up the contrast agent and make normal parenchyma brighter on T1‐weighted images while tumour cells do not take up the contrast agent, hence increasing the contrast between focal lesions and normal liver tissue. However, it has also been shown that the relative signal enhancement following injection of gadoxetate disodium is reduced in patients with fibrosis.^76^, ^77^, ^78^ This could be explained by two possible mechanisms:

- dilution of functioning hepatocytes by presence of fibrosis
- reduced uptake into the hepatocytes by reduced OATP1 action

It has also been shown that gadoxetate disodium uptake is reduced also in subjects with hepatic inflammation and ballooning.^76^ It could therefore be hypothesized that anything that should not be present in the liver, for example fibrosis and inflammatory cells, will dilute the concentration of functional hepatocytes and that the conditions associated with fibrosis and inflammation, for example oxidative stress and mitochondrial dysfunction, also will affect the transporters associated with gadoxetate disodium uptake and excretion.

Several studies have been performed utilizing dynamic imaging with gadoxetate disodium,^79^, ^80^ and this allows quantitative information to be extracted by compartmental modelling yielding data on uptake and excretion rate of gadoxetate disodium in the hepatocytes but also quantitative assessment of extracellular volume in the liver, the same parameter that is associated with T1 but here directly quantified. Emerging data recently presented at the International Liver Congress in 2019 have assessed the predictive value of gadoxetate disodium imaging in patients with compensated (N = 110) and decompensated (N = 99) cirrhosis and have reported good prognostic information in both groups with a follow‐up time of 48 months.^81^ However, the following drawbacks need to be considered. The use of Gadoxetate Disodium is currently not indicated for patients with NAFLD, furthermore the use of a Gadolinium‐based contrast agent may carry an increased risk with potential retention of gadolinium in the body^82^ and are contraindicated in subjects with GFR < 30, and finally there are currently no interventional data in humans available using gadoxetate disodium in NAFLD and NASH.

Other functional methods assessing the liver include quantification of portal flow using phase‐contrast MRI and portal pulsatility as a measure of vascular resistance in the liver using MRI. As described previously, the concept of stress testing is readily available in the diagnostic workup in other disease areas but not for NAFLD. There are however possibilities to combine MRI‐based methods such as gadoxetate disodium imaging or 31P‐MRS of ATP with functional challenge, for example fructose, to determine the functional reserve capacity of the liver to better understand the prognosis of a patient or effects of a pharmacological treatment. These methods are however not readily available, and further studies are needed to understand and validate the utility of these methods.

## 6 CONCLUSION

MRI is today an important tool in research of NAFLD and NASH patients. The most commonly used methods include PDFF, MRE and T1 measurements. Out of these, PDFF is well validated and frequently used and gives a more reproducible assessment of liver steatosis than liver biopsy. MRE, T1 measurements and functional MRI are also used in interventional studies but more data on relation between treatments effects seen with MRI/MRE and biopsy is required to fully understand the utility in clinical research. All these methods can also be used in the clinical workup of patients, and again here, PDFF is the best validated method. Larger prospective studies using some of the methods described in this paper are underway and will guide us about the clinical utility.

## CONFLICT OF INTERESTS

C.C reports no conflict of interests, and L.J is an employee and shareholder of Antaros Medical.

## AUTHOR CONTRIBUTIONS

Cyrielle Caussy drafted the manuscript, involved in critical revision of the manuscript and approved final submission. Lars Johansson drafted the manuscript, involved in critical revision of the manuscript and approved final submission. All authors approved the final version of this article.

## ETHICAL APPROVAL

This is a review paper; no separate informed consent has been obtained to write this.

## Data Availability

No new data generated in this review article.

## References

[1] Loomba R , Sanyal AJ . The global NAFLD epidemic. Nat Rev Gastroenterol Hepatol. 2013;10(11):686‐690. 24042449 10.1038/nrgastro.2013.171

[2] Younossi ZM , Koenig AB , Abdelatif D , Fazel Y , Henry L , Wymer M . Global epidemiology of nonalcoholic fatty liver disease‐meta‐analytic assessment of prevalence, incidence, and outcomes. Hepatology. 2016;64(1):73‐84. 26707365 10.1002/hep.28431

[3] Younossi Z , Anstee QM , Marietti M , et al. Global burden of NAFLD and NASH: trends, predictions, risk factors and prevention. Nat Rev Gastroenterol Hepatol. 2018;15(1):11‐20. 28930295 10.1038/nrgastro.2017.109

[4] Dulai PS , Singh S , Patel J , et al. Increased risk of mortality by fibrosis stage in nonalcoholic fatty liver disease: systematic review and meta‐analysis. Hepatology. 2017;65(5):1557‐1565. 28130788 10.1002/hep.29085 PMC5397356

[5] Charlton MR , Burns JM , Pedersen RA , Watt KD , Heimbach JK , Dierkhising RA . Frequency and outcomes of liver transplantation for nonalcoholic steatohepatitis in the United States. Gastroenterology. 2011;141(4):1249‐1253. 21726509 10.1053/j.gastro.2011.06.061

[6] Vernon G , Baranova A , Younossi ZM . Systematic review: the epidemiology and natural history of non‐alcoholic fatty liver disease and non‐alcoholic steatohepatitis in adults. Aliment Pharmacol Ther. 2011;34(3):274‐285. 21623852 10.1111/j.1365-2036.2011.04724.x

[7] Wong RJ , Aguilar M , Cheung R , et al. Nonalcoholic steatohepatitis is the second leading etiology of liver disease among adults awaiting liver transplantation in the United States. Gastroenterology. 2015;148(3):547‐555. 25461851 10.1053/j.gastro.2014.11.039

[8] Younossi Z , Ratziu V , Loomba R , et al. GS‐06‐positive results from REGENERATE: a phase 3 International, Randomized, Placebo‐Controlled Study evaluating obeticholic acid treatment for NASH. J Hepatol. 2019;70(1):e5.

[9] Younossi ZM , Loomba R , Rinella ME , et al. Current and future therapeutic regimens for nonalcoholic fatty liver disease and nonalcoholic steatohepatitis. Hepatology. 2018;68(1):361‐371. 29222911 10.1002/hep.29724 PMC6508084

[10] Angulo P . Nonalcoholic fatty liver disease. N Engl J Med. 2002;346(16):1221‐1231. 11961152 10.1056/NEJMra011775

[11] Asrani SK . Incorporation of noninvasive measures of liver fibrosis into clinical practice: diagnosis and prognosis. Clin Gastroenterol Hepatol. 2015;13(12):2190‐2204. 26226095 10.1016/j.cgh.2015.07.030

[12] Bravo AA , Sheth SG , Chopra S . Liver biopsy. N Engl J Med. 2001;344(7):495‐500. 11172192 10.1056/NEJM200102153440706

[13] Regev A , Berho M , Jeffers LJ , et al. Sampling error and intraobserver variation in liver biopsy in patients with chronic HCV infection. Am J Gastroenterol. 2002;97(10):2614‐2618. 12385448 10.1111/j.1572-0241.2002.06038.x

[14] Rockey DC , Caldwell SH , Goodman ZD , Nelson RC , Smith AD . Liver biopsy. Hepatology. 2009;49(3):1017‐1044. 19243014 10.1002/hep.22742

[15] Sumida Y , Nakajima A , Itoh Y . Limitations of liver biopsy and non‐invasive diagnostic tests for the diagnosis of nonalcoholic fatty liver disease/nonalcoholic steatohepatitis. World J Gastroenterol. 2014;20(2):475‐485. 24574716 10.3748/wjg.v20.i2.475 PMC3923022

[16] Loomba R . Role of imaging‐based biomarkers in NAFLD: Recent advances in clinical application and future research directions. J Hepatol. 2018;68(2):296‐304. 29203392 10.1016/j.jhep.2017.11.028 PMC5810949

[17] Vilar‐Gomez E , Chalasani N . Non‐invasive assessment of non‐alcoholic fatty liver disease: clinical prediction rules and blood‐based biomarkers. J Hepatol. 2018;68(2):305‐315. 29154965 10.1016/j.jhep.2017.11.013

[18] Chalasani N , Younossi Z , Lavine JE , et al. The diagnosis and management of nonalcoholic fatty liver disease: practice guidance from the American Association for the Study of Liver Diseases. Hepatology. 2018;67(1):328‐357. 28714183 10.1002/hep.29367

[19] Schwenzer NF , Springer F , Schraml C , Stefan N , Machann J , Schick F . Non‐invasive assessment and quantification of liver steatosis by ultrasound, computed tomography and magnetic resonance. J Hepatol. 2009;51(3):433‐445. 19604596 10.1016/j.jhep.2009.05.023

[20] Szczepaniak LS , Nurenberg P , Leonard D , et al. Magnetic resonance spectroscopy to measure hepatic triglyceride content: prevalence of hepatic steatosis in the general population. Am J Physiol Endocrinol Metab. 2005;288(2):E462‐E468. 15339742 10.1152/ajpendo.00064.2004

[21] Tang A , Tan J , Sun M , et al. Nonalcoholic fatty liver disease: MR imaging of liver proton density fat fraction to assess hepatic steatosis. Radiology. 2013;267(2):422‐431. 23382291 10.1148/radiol.12120896 PMC3632805

[22] Reeder SB , Hu HH , Sirlin CB . Proton density fat‐fraction: a standardized MR‐based biomarker of tissue fat concentration. J Magn Reson Imaging. 2012;36(5):1011‐1014. 22777847 10.1002/jmri.23741 PMC4779595

[23] Bonekamp S , Tang A , Mashhood A , et al. Spatial distribution of MRI‐Determined hepatic proton density fat fraction in adults with nonalcoholic fatty liver disease. J Magn Reson Imaging. 2014;39(6):1525‐1532. 24987758 10.1002/jmri.24321 PMC4083476

[24] Kang GH , Cruite I , Shiehmorteza M , et al. Reproducibility of MRI‐determined proton density fat fraction across two different MR scanner platforms. J Magn Reson Imaging. 2011;34(4):928‐934. 21769986 10.1002/jmri.22701 PMC4803481

[25] Negrete LM , Middleton MS , Clark L , et al. Inter‐examination precision of magnitude‐based MRI for estimation of segmental hepatic proton density fat fraction in obese subjects. J Magn Reson Imaging. 2014;39(5):1265‐1271. 24136736 10.1002/jmri.24284 PMC3984359

[26] Noureddin M , Lam J , Peterson MR , et al. Utility of magnetic resonance imaging versus histology for quantifying changes in liver fat in nonalcoholic fatty liver disease trials. Hepatology. 2013;58(6):1930‐1940. 23696515 10.1002/hep.26455 PMC4819962

[27] Yokoo T , Shiehmorteza M , Hamilton G , et al. Estimation of hepatic proton‐density fat fraction by using MR imaging at 3.0 T. Radiology. 2011;258(3):749‐759. 21212366 10.1148/radiol.10100659 PMC3042639

[28] Middleton MS , Van Natta ML , Heba ER , et al. Diagnostic accuracy of magnetic resonance imaging hepatic proton density fat fraction in pediatric nonalcoholic fatty liver disease. Hepatology. 2018;67(3):858‐872. 29028128 10.1002/hep.29596 PMC6211296

[29] Hines CD , Frydrychowicz A , Hamilton G , et al. T(1) independent, T(2) (*) corrected chemical shift based fat‐water separation with multi‐peak fat spectral modeling is an accurate and precise measure of hepatic steatosis. J Magn Reson Imaging. 2011;33(4):873‐881. 21448952 10.1002/jmri.22514 PMC3130738

[30] Meisamy S , Hines CD , Hamilton G , et al. Quantification of hepatic steatosis with T1‐independent, T2‐corrected MR imaging with spectral modeling of fat: blinded comparison with MR spectroscopy. Radiology. 2011;258(3):767‐775. 21248233 10.1148/radiol.10100708 PMC3042638

[31] Rehm JL , Wolfgram PM , Hernando D , Eickhoff JC , Allen DB , Reeder SB . Proton density fat‐fraction is an accurate biomarker of hepatic steatosis in adolescent girls and young women. Eur Radiol. 2015;25(10):2921‐2930. 25916386 10.1007/s00330-015-3724-1 PMC4564339

[32] Yokoo T , Bydder M , Hamilton G , et al. Nonalcoholic fatty liver disease: diagnostic and fat‐grading accuracy of low‐flip‐angle multiecho gradient‐recalled‐echo MR imaging at 1.5 T. Radiology. 2009;251(1):67‐76. 19221054 10.1148/radiol.2511080666 PMC2663579

[33] Permutt Z , Le TA , Peterson MR , et al. Correlation between liver histology and novel magnetic resonance imaging in adult patients with non‐alcoholic fatty liver disease – MRI accurately quantifies hepatic steatosis in NAFLD. Aliment Pharmacol Ther. 2012;36(1):22‐29. 22554256 10.1111/j.1365-2036.2012.05121.x PMC3437221

[34] Tang A , Desai A , Hamilton G , et al. Accuracy of MR imaging‐estimated proton density fat fraction for classification of dichotomized histologic steatosis grades in nonalcoholic fatty liver disease. Radiology. 2015;274(2):416‐425. 25247408 10.1148/radiol.14140754 PMC4314291

[35] Yokoo T , Serai SD , Pirasteh A , et al. Linearity, bias, and precision of hepatic proton density fat fraction measurements by using MR imaging: a meta‐analysis. Radiology. 2018;286(2):486‐498. 28892458 10.1148/radiol.2017170550 PMC5813433

[36] Loomba R , Sirlin CB , Ang B , et al. Ezetimibe for the treatment of nonalcoholic steatohepatitis: assessment by novel magnetic resonance imaging and magnetic resonance elastography in a randomized trial (MOZART trial). Hepatology. 2015;61(4):1239‐1250. 25482832 10.1002/hep.27647 PMC4407930

[37] Middleton MS , Heba ER , Hooker CA , et al. Agreement between magnetic resonance imaging proton density fat fraction measurements and pathologist‐assigned steatosis grades of liver biopsies from adults with nonalcoholic steatohepatitis. Gastroenterology. 2017;153(3):753‐761. 28624576 10.1053/j.gastro.2017.06.005 PMC5695870

[38] Cui J , Philo L , Nguyen P , et al. Sitagliptin vs. placebo for non‐alcoholic fatty liver disease: a randomized controlled trial. J Hepatol. 2016;65(2):369‐376. 27151177 10.1016/j.jhep.2016.04.021 PMC5081213

[39] Tyagi A , Yeganeh O , Levin Y , et al. Intra‐ and inter‐examination repeatability of magnetic resonance spectroscopy, magnitude‐based MRI, and complex‐based MRI for estimation of hepatic proton density fat fraction in overweight and obese children and adults. Abdom Imaging. 2015;40(8):3070‐3077. 26350282 10.1007/s00261-015-0542-5 PMC4819976

[40] Le TA , Chen J , Changchien C , et al. Effect of colesevelam on liver fat quantified by magnetic resonance in nonalcoholic steatohepatitis: a randomized controlled trial. Hepatology. 2012;56(3):922‐932. 22431131 10.1002/hep.25731 PMC3400720

[41] Patel NS , Doycheva I , Peterson MR , et al. Effect of weight loss on magnetic resonance imaging estimation of liver fat and volume in patients with nonalcoholic steatohepatitis. Clin Gastroenterol Hepatol. 2015;13(3):561‐568.e1. 25218667 10.1016/j.cgh.2014.08.039 PMC4333065

[42] Oscarsson J , Onnerhag K , Riserus U , et al. Effects of free omega‐3 carboxylic acids and fenofibrate on liver fat content in patients with hypertriglyceridemia and non‐alcoholic fatty liver disease: a double‐blind, randomized, placebo‐controlled study. J Clin Lipidol. 2018;12(6):1390‐1403.e4. 30197273 10.1016/j.jacl.2018.08.003

[43] Caussy C , Reeder SB , Sirlin CB , Loomba R . Noninvasive, quantitative assessment of liver fat by MRI‐PDFF as an endpoint in NASH trials. Hepatology. 2018;68(2):763‐772. 29356032 10.1002/hep.29797 PMC6054824

[44] Patel J , Bettencourt R , Cui J , et al. Association of noninvasive quantitative decline in liver fat content on MRI with histologic response in nonalcoholic steatohepatitis. Therap Adv Gastroenterol. 2016;9(5):692‐701. 10.1177/1756283X16656735 PMC4984335 27582882

[45] Ajmera V , Park CC , Caussy C , et al. Magnetic resonance imaging proton density fat fraction associates with progression of fibrosis in patients with nonalcoholic fatty liver disease. Gastroenterology. 2018;155(2):307‐310.e2. 29660324 10.1053/j.gastro.2018.04.014 PMC6090543

[46] Angulo P , Kleiner DE , Dam‐Larsen S , et al. Liver fibrosis, but no other histologic features, is associated with long‐term outcomes of patients with nonalcoholic fatty liver disease. Gastroenterology. 2015;149(2):389‐397.e10 25935633 10.1053/j.gastro.2015.04.043 PMC4516664

[47] Ekstedt M , Hagstrom H , Nasr P , et al. Fibrosis stage is the strongest predictor for disease‐specific mortality in NAFLD after up to 33 years of follow‐up. Hepatology. 2015;61(5):1547‐1554. 25125077 10.1002/hep.27368

[48] Tapper EB , Loomba R . Noninvasive imaging biomarker assessment of liver fibrosis by elastography in NAFLD. Nat Rev Gastroenterol Hepatol. 2018;15(5):274‐282. 29463906 10.1038/nrgastro.2018.10 PMC7504909

[49] Chen J , Yin M , Talwalkar JA , et al. Diagnostic performance of MR elastography and vibration‐controlled transient elastography in the detection of hepatic fibrosis in patients with severe to morbid obesity. Radiology. 2017;283(2):418‐428. 27861111 10.1148/radiol.2016160685 PMC5395333

[50] Venkatesh SK , Yin M , Ehman RL . Magnetic resonance elastography of liver: technique, analysis, and clinical applications. J Magn Reson Imaging. 2013;37(3):544‐555. 23423795 10.1002/jmri.23731 PMC3579218

[51] Yin M , Glaser KJ , Talwalkar JA , Chen J , Manduca A , Ehman RL . Hepatic MR elastography: clinical performance in a series of 1377 consecutive examinations. Radiology. 2016;278(1):114‐124. 26162026 10.1148/radiol.2015142141 PMC4688072

[52] Cui J , Ang B , Haufe W , et al. Comparative diagnostic accuracy of magnetic resonance elastography vs. eight clinical prediction rules for non‐invasive diagnosis of advanced fibrosis in biopsy‐proven non‐alcoholic fatty liver disease: a prospective study. Aliment Pharmacol Ther. 2015;41(12):1271‐1280. 25873207 10.1111/apt.13196 PMC4532628

[53] Caussy C , Chen J , Alquiraish MH , et al. Association between obesity and discordance in fibrosis stage determination by magnetic resonance vs transient elastography in patients with nonalcoholic liver disease. Clin Gastroenterol Hepatol. 2018;16(12):1974‐1982.e7. 29104128 10.1016/j.cgh.2017.10.037 PMC6050151

[54] Cui J , Heba E , Hernandez C , et al. Magnetic resonance elastography is superior to acoustic radiation force impulse for the diagnosis of fibrosis in patients with biopsy‐proven nonalcoholic fatty liver disease: a prospective study. Hepatology. 2016;63(2):453‐461. 26560734 10.1002/hep.28337 PMC4818645

[55] Hsu C , Caussy C , Imajo K , et al. Magnetic resonance vs transient elastography analysis of patients with nonalcoholic fatty liver disease: a systematic review and pooled analysis of individual participants. Clin Gastroenterol Hepatol. 2019;17(4):630‐637.e8. 29908362 10.1016/j.cgh.2018.05.059 PMC6294709

[56] Wagner M , Corcuera‐Solano I , Lo G , et al. Technical failure of MR elastography examinations of the liver: Experience from a Large Single‐Center Study. Radiology. 2017;284(2):401‐412. 28045604 10.1148/radiol.2016160863 PMC5548447

[57] Imajo K , Kessoku T , Honda Y , et al. Magnetic resonance imaging more accurately classifies steatosis and fibrosis in patients with nonalcoholic fatty liver disease than transient elastography. Gastroenterology. 2016;150(3):626‐637.e7. 26677985 10.1053/j.gastro.2015.11.048

[58] Park CC , Nguyen P , Hernandez C , et al. Magnetic resonance elastography vs transient elastography in detection of fibrosis and noninvasive measurement of steatosis in patients with biopsy‐proven nonalcoholic fatty liver disease. Gastroenterology. 2017;152(3):598‐607. 27911262 10.1053/j.gastro.2016.10.026 PMC5285304

[59] Loomba R , Wolfson T , Ang B , et al. Magnetic resonance elastography predicts advanced fibrosis in patients with nonalcoholic fatty liver disease: a prospective study. Hepatology. 2014;60(6):1920‐1928. 25103310 10.1002/hep.27362 PMC4245360

[60] Harrison SA , Dennis A , Fiore MM , et al. Utility and variability of three non‐invasive liver fibrosis imaging modalities to evaluate efficacy of GR‐MD‐02 in subjects with NASH and bridging fibrosis during a phase‐2 randomized clinical trial. PLoS ONE. 2018;13(9):e0203054. 30192782 10.1371/journal.pone.0203054 PMC6128474

[61] Jayakumar S , Middleton MS , Lawitz EJ , et al. Longitudinal correlations between MRE, MRI‐PDFF, and liver histology in patients with non‐alcoholic steatohepatitis: analysis of data from a phase II trial of selonsertib. J Hepatol. 2019;70(1):133‐141. 30291868 10.1016/j.jhep.2018.09.024

[62] Patel NS , Hooker J , Gonzalez M , et al. Weight loss decreases magnetic resonance elastography estimated liver stiffness in nonalcoholic fatty liver disease. Clin Gastroenterol Hepatol. 2017;15(3):463‐464. 27712981 10.1016/j.cgh.2016.09.150 PMC5476292

[63] Sanyal A , Charles ED , Neuschwander‐Tetri BA , et al. Pegbelfermin (BMS‐986036), a PEGylated fibroblast growth factor 21 analogue, in patients with non‐alcoholic steatohepatitis: a randomised, double‐blind, placebo‐controlled, phase 2a trial. Lancet. 2019;392(10165):2705‐2717. 30554783 10.1016/S0140-6736(18)31785-9

[64] Loomba R , Lawitz E , Ghalib R , et al. Longitudinal changes in liver stiffness by magnetic resonance elastography (MRE), liver fibrosis, and serum markers of fibrosis in a multi‐center clinical trial in nonalcoholic steatohepatitis (NASH). J Hepatol. 2017;66:S671.

[65] Ajmera VH , Liu A , Singh S , et al. Clinical utility of an increase in magnetic resonance elastography in predicting fibrosis progression in NAFLD. Hepatology. 2020;71:849‐860. 31556124 10.1002/hep.30974 PMC7828573

[66] Loomba R , Cui J , Wolfson T , et al. Novel 3D magnetic resonance elastography for the noninvasive diagnosis of advanced fibrosis in NAFLD: a prospective study. Am J Gastroenterol. 2016;111(7):986‐994. 27002798 10.1038/ajg.2016.65 PMC5001170

[67] Smith FW , Mallard JR , Reid A , Hutchison JM . Nuclear magnetic resonance tomographic imaging in liver disease. Lancet. 1981;1(8227):963‐966. 6112385 10.1016/s0140-6736(81)91731-1

[68] Heye T , Yang SR , Bock M , et al. MR relaxometry of the liver: significant elevation of T1 relaxation time in patients with liver cirrhosis. Eur Radiol. 2012;22(6):1224‐1232. 22302503 10.1007/s00330-012-2378-5

[69] Banerjee R , Pavlides M , Tunnicliffe EM , et al. Multiparametric magnetic resonance for the non‐invasive diagnosis of liver disease. J Hepatol. 2014;60(1):69‐77. 24036007 10.1016/j.jhep.2013.09.002 PMC3865797

[70] Pavlides M , Banerjee R , Tunnicliffe EM , et al. Multiparametric magnetic resonance imaging for the assessment of non‐alcoholic fatty liver disease severity. Liver Int. 2017;37(7):1065‐1073. 27778429 10.1111/liv.13284 PMC5518289

[71] Pavlides M , Banerjee R , Sellwood J , et al. Multiparametric magnetic resonance imaging predicts clinical outcomes in patients with chronic liver disease. J Hepatol. 2016;64(2):308‐315. 26471505 10.1016/j.jhep.2015.10.009 PMC4751288

[72] Mojtahed A , Kelly CJ , Herlihy AH , et al. Reference range of liver corrected T1 values in a population at low risk for fatty liver disease‐a UK Biobank sub‐study, with an appendix of interesting cases. Abdom Radiol (NY). 2019;44(1):72‐84. 30032383 10.1007/s00261-018-1701-2 PMC6348264

[73] Heymsfield SB , Thomas D , Nguyen AM , et al. Voluntary weight loss: systematic review of early phase body composition changes. Obes Rev. 2011;12(5):e348‐e361. 20524998 10.1111/j.1467-789X.2010.00767.x

[74] Bashir MR , Wolfson T , Gamst AC , et al. Hepatic R2* is more strongly associated with proton density fat fraction than histologic liver iron scores in patients with nonalcoholic fatty liver disease. J Magn Reson Imaging. 2019;49(5):1456‐1466. 30318834 10.1002/jmri.26312 PMC6449180

[75] Allen AM , Shah VH , Therneau TM , et al. The role of three‐dimensional magnetic resonance elastography in the diagnosis of nonalcoholic steatohepatitis in obese patients undergoing bariatric surgery. Hepatology. 2020;71(2):510‐521. 30582669 10.1002/hep.30483 PMC6591099

[76] Bastati N , Feier D , Wibmer A , et al. Noninvasive differentiation of simple steatosis and steatohepatitis by using gadoxetic acid‐enhanced MR imaging in patients with nonalcoholic fatty liver disease: a proof‐of‐concept study. Radiology. 2014;271(3):739‐747. 24576046 10.1148/radiol.14131890

[77] Li X , Liu H , Wang R , Yang J , Zhang Y , Li C . Gadoxetate‐disodium‐enhanced magnetic resonance imaging for liver fibrosis staging: a systematic review and meta‐analysis. Clin Radiol. 2020;75:319. 10.1016/j.crad.2019.11.001 31831141

[78] Verloh N , Utpatel K , Haimerl M , et al. Liver fibrosis and Gd‐EOB‐DTPA‐enhanced MRI: a histopathologic correlation. Sci Rep. 2015;5:15408. 26478097 10.1038/srep15408 PMC5378898

[79] Juluru K , Talal AH , Yantiss RK , et al. Diagnostic accuracy of intracellular uptake rates calculated using dynamic Gd‐EOB‐DTPA‐enhanced MRI for hepatic fibrosis stage. J Magn Reson Imaging. 2017;45(4):1177‐1185. 27527820 10.1002/jmri.25431 PMC5313385

[80] Leporq B , Daire JL , Pastor CM , et al. Quantification of hepatic perfusion and hepatocyte function with dynamic gadoxetic acid‐enhanced MRI in patients with chronic liver disease. Clin Sci (Lond). 2018;132(7):813‐824. 29440620 10.1042/CS20171131

[81] Mandorfer M , Bastati N , Beer L , et al. Functional Liver Imaging Score Derived from Gadoxetic Acid‐Enhanced MRI Predicts Outcomes in Patients with Advanced Chronic Liver Disease. Paper presented at the International Liver Congress Vienna, Austria.

[82] McDonald RJ , Levine D , Weinreb J , et al. Gadolinium retention: a research roadmap from the 2018 NIH/ACR/RSNA Workshop on gadolinium chelates. Radiology. 2018;289(2):517‐534. 30204075 10.1148/radiol.2018181151 PMC6209069

